# REM Sleep Preserves Affective Response to Social Stress—Experimental Study

**DOI:** 10.1523/ENEURO.0453-23.2024

**Published:** 2024-06-03

**Authors:** Risto Halonen, Liisa Kuula, Maikki Selin, Alma Suutari, Minea Antila, Anu-Katriina Pesonen

**Affiliations:** SleepWell Research Program Unit, Faculty of Medicine, University of Helsinki, Helsinki FI-00014, Finland

**Keywords:** affective regulation, REM sleep, sleep suppression, slow-wave sleep, SO–spindle coupling, theta power

## Abstract

Sleep's contribution to affective regulation is insufficiently understood. Previous human research has focused on memorizing or rating affective pictures and less on physiological affective responsivity. This may result in overlapping definitions of affective and declarative memories and inconsistent deductions for how rapid eye movement sleep (REMS) and slow-wave sleep (SWS) are involved. Literature associates REMS theta (4–8 Hz) activity with emotional memory processing, but its contribution to social stress habituation is unknown. Applying selective sleep stage suppression and oscillatory analyses, we investigated how sleep modulated affective adaptation toward social stress and retention of neutral declarative memories. Native Finnish participants (*N* = 29; age, *M* = 25.8 years) were allocated to REMS or SWS suppression conditions. We measured physiological (skin conductance response, SCR) and subjective stress response and declarative memory retrieval thrice: before laboratory night, the next morning, and after 3 d. Linear mixed models were applied to test the effects of condition and sleep parameters on emotional responsivity and memory retrieval. Greater overnight increase in SCR toward the stressor emerged after suppressed SWS (intact REMS) relative to suppressed REMS (20.1% vs 6.1%; *p* = 0.016). The overnight SCR increase was positively associated with accumulated REMS theta energy irrespective of the condition (*r* = 0.601; *p* = 0.002). Subjectively rated affective response and declarative memory recall were comparable between the conditions. The contributions of REMS and SWS to habituation of social stress are distinct. REMS theta activity proposedly facilitates the consolidation of autonomic affective responses. Declarative memory consolidation may not have greater dependence on intact SWS relative to intact REMS.

## Significance Statement

Disrupted sleep is a common problem with negative effects on affective regulation. While research indicates that rapid eye movement sleep (REMS) has a central role in off-line affective processing, the mechanisms are not well defined. We used selective sleep stage suppression to investigate how disrupted sleep- and stage-specific neural activity modulated the affective responsivity toward a self-conscious stressor inducing shame. We show that theta band oscillatory activity during REMS is especially important for preserving the physiological stress response overnight. Understanding sleep-driven affective regulation facilitates development of applications aiming at improving mental well-being.

## Introduction

Sleep stages contribute distinctively to processing recent experiences and memories. Nonrapid eye movement sleep (NREMS) and especially its deepest stage, slow-wave sleep (SWS), facilitate declarative memory consolidation via synchronized occurrence of slow oscillations (SOs) and sleep spindles (Klinzing et al., 2019). On the other hand, emotional processing is often attributed to rapid eye movement sleep (REMS). According to the sleep-to-remember, sleep-to-forget (SRSF) hypothesis, REMS contributes specifically to the depotentiation of the emotional charge linked to memories (i.e., “forgetting”; Walker and van der Helm, 2009). This dilution of potentially distressing experiences has been suggested to be important for mental health (Wassing et al., 2016), and some empirical evidence supports the hypothesis (Gujar et al., 2011; Rosales-Lagarde et al., 2012). However, a number of studies claim the opposite, i.e., that REMS preserves the affective component of memories (Lara-Carrasco et al., 2009; Pace-Schott et al., 2011; Baran et al., 2012; Werner et al., 2015, 2021). To better understand emotional regulation during REMS, further experimental studies with variable affective induction and active manipulation of potential mechanisms are warranted.

A powerful experimental method for disentangling sleep stage-specific effects is selective sleep stage deprivation/suppression. In human studies, suppressing nocturnal REMS induced increased amygdala response toward social exclusion (Glosemeyer et al., 2020), impaired fear extinction (Spoormaker et al., 2012), and habituation to threatening visual stimuli (Rosales-Lagarde et al., 2012). However, contrasting findings also exist. For instance, REMS deprivation decreased subjective arousal ratings toward aversive pictures the next morning (Lara-Carrasco et al., 2009; Werner et al., 2021), although in one study, the short-term effect was inverted after 2 d (Werner et al., 2021).

Most of these earlier suppression studies have examined memory retention, or subjective rating, of affective pictures, leaving it open how the physiological stress response is modulated during sleep. There is little evidence on how sleep explains the adaptation to, or preservation of, physiological affective response in humans. Only one study (Spoormaker et al., 2012) investigated how sleep stage suppression influenced physiological stress response. In that study, REMS-deprived participants showed impaired fear memory extinction reflected by increased electrodermal response. In addition, previous sleep suppression studies have not examined how neural activity markers in sleep, such as spectral power in electroencephalography (EEG), influence emotional reactivity overnight.

Animal models have provided evidence that EEG dynamics predict affective processing in intact sleep. Intracortical recordings in rats suggest that especially theta (∼4–8 Hz) oscillations during REMS stand out in this regard. For instance, increased REMS theta coherence between the amygdala and hippocampus or medial prefrontal cortex predicted stronger fear memory consolidation (Popa et al., 2010). Another study found that REMS theta activity increased after successful avoidance task training (Fogel et al., 2009). Theta activity is proposedly linked to pontine–geniculo–occipital waves (Hutchison and Rathore, 2015) reflecting hippocampal–amygdala synchrony (Karashima et al., 2010) and promoting synaptic plasticity processes and emotional learning (Mavanji et al., 2004; Datta et al., 2008). Pontine–geniculo–occipital waves are unmeasurable in humans with scalp EEG. In human studies, EEG-measured REMS theta power (Prehn-Kristensen et al., 2013; Kim et al., 2020) and its right-hemisphere lateralization (Nishida et al., 2009; Sopp et al., 2017) have predicted better retention of especially emotional memory content.

To delineate sleep's impact on emotional processing, an experiment should also assess declarative memory. Declarative memory consolidation depends on the oscillatory components of NREMS, particularly SWS (Léger et al., 2018; Klinzing et al., 2019). Numerous studies have shown that sleep spindles (Clemens et al., 2005) and their coupling to SOs (Helfrich et al., 2018; Mikutta et al., 2019; Muehlroth et al., 2019; Halonen, Kuula, Antila, et al., 2021; Weiner et al., 2024) predict memory retention. Given this, it is rather unexpected that selective suppressing of SWS has not conclusively resulted in impaired memory retention in previous studies. For example, the retention of verbal (Genzel et al., 2009; Casey et al., 2016) or visual (Wiesner et al., 2015) memories was not differentially affected by SWS or REMS suppression. In one study, however, suppressed SWS impaired visuospatial memory (Casey et al., 2016). In these studies, either sleep spindles did not predict memory outcome (Casey et al., 2016), or their relevance in learning was N2-specific (Genzel et al., 2009). Examination of SO–spindle coupling in sleep suppression studies is lacking overall.

In an earlier study, a higher overnight REMS percentage predicted preserved physiological response to social stress-related shame (Halonen, Kuula, Makkonen, et al., 2021). The current study went further to investigate how selective sleep stage suppression, REMS or SWS, affected habituation to a stressor inducing self-conscious affect. To better delineate the affective processing component, we also included a declarative memory task. For that purpose, we applied a memory paradigm using novel metaphors, which has been shown to depend on SO–spindle coupling in SWS and N2 (Halonen, Kuula, Antila, et al., 2021).

We assumed that manipulating REMS would impact off-line affective processing and that the overnight change would be associated with REMS theta activity. However, the direction of the effect cannot be predicted based on existing human studies. Regarding declarative memory, we did not expect suppression effects. Instead, we assumed that SO-coupled spindle events would positively predict postsleep performance and that N2 would compensate for reduced SWS coupling events.

## Materials and Methods

### Participants

The sample consisted of 29 young adults (14 males, 15 females, self-disclosed; age, *M* = 25.8 years; SD = 4.5 years; range, 19.0–36.4 years) living in the capital area of Finland. Seven participants were psychology undergraduates, thirteen were recruited from academic hobby societies, and nine were recruited from a local sports club. Compensation of €100 was provided to all participants. Measurements were performed in May–November 2021. The participants answered questionnaires for background information and physical and mental well-being. None of the participants met the exclusion criteria (severe anxiety, depression or sleep disorders, or major neurological conditions). Participants were randomly allocated to either REMS suppression (REMS_SUPPR_; *n* = 15) or SWS suppression (SWS_SUPPR_; *n* = 14) condition, however, such that sex distribution did not differ between the conditions. Participants were blind to the condition.

All participants provided written informed consents prior to participation. The study protocol was approved by Helsinki University Hospital Ethics Committee (HUS/1390/2019), and all components of the study were conducted in accordance with the Declaration of Helsinki and its later amendments.

### Study flow and sleep suppression

**Figure 1.**
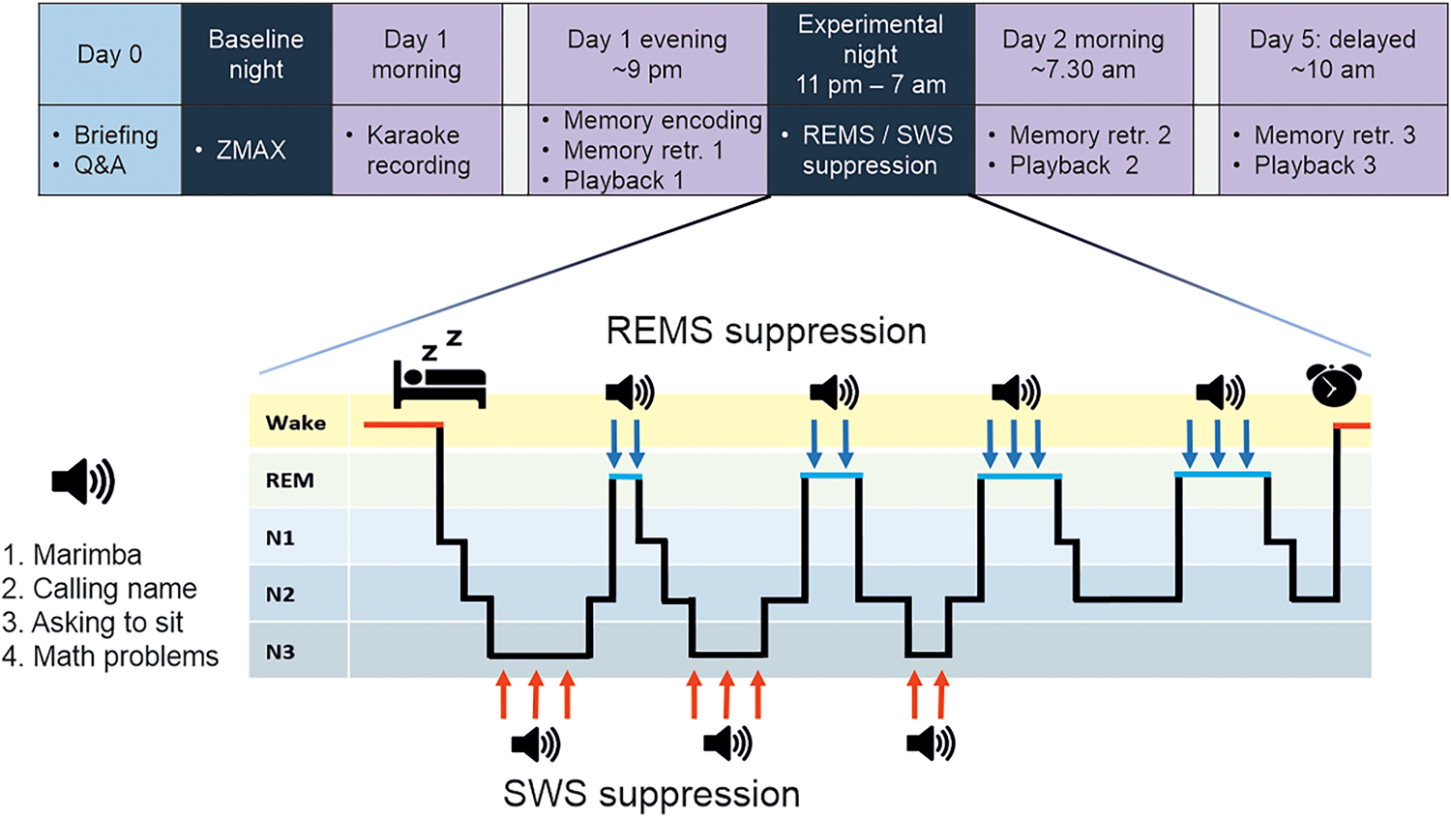
An overview of the study process and the suppression protocol. During the experimental night, sleep was disrupted when participants entered the sleep stage of their respective suppression condition (REMS_SUPPR_ / SWS_SUPPR_). In all playbacks, participants' own singing was played to them via loudspeakers, and physiological and subjective stress responses were measured. In memory retrievals, a subset of encoded metaphors was tested with cued recall.

Participants were asked to maintain a regular sleep rhythm for 3 d prior to the study. On Day 0 (Fig. 1), participants visited the laboratory to obtain a ZMAX device (Hypnodyne) for in-home sleep recording (Baseline night). Briefing was given via Zoom. The following day (Day 1), at the laboratory, participants sang a karaoke version of Abba's Dancing Queen, which was recorded. After spending the day in their normal daily routines, the participants arrived at the laboratory at 9 P.M. and underwent memory encoding, the first karaoke playback and finally the first metaphor retrieval. The evening test session took ∼45 min. Polysomnography (PSG) was setup. Sleep opportunity was given between 11 P.M. and 7 A.M. Depending on the suppression condition (REMS_SUPPR_ / SWS_SUPPR_), the research assistant woke up the participant when distinct REMS-related rapid eye movements or obvious SWS (i.e., the majority of an epoch consisting of approximately <∼1 Hz oscillations) were detected on the real-time PSG. The awakenings were done via in-room loudspeaker with progressive intensity: (1) a melody (marimba), (2) calling the participant's name, (3) asking to sit, or (4) presenting simple math problems. If the participant did not wake up with, e.g., marimba, or drifted to the to-be-suppressed sleep stage in a few minutes, a higher intensity step (e.g., calling their name) was applied. On Day 2 morning (>30 min after awakening), a second memory retrieval and karaoke playback took place. On Day 5 (∼10 A.M.), the final metaphor retrieval and karaoke playback were administered (Fig. 1). The test sessions on Day 2 and Day 5 took ∼25 min each.

### Memory task

The memory task was run with the Presentation software 22.0 (Neurobehavioral Systems). The task material consisted of 60 novel verbal metaphors, obtained from a study examining psycholinguistic dimensions (Herkman and Servie, 2013). The metaphors were divided into two subsets of 30 metaphors each: pre- and postsleep recall. We matched the subsets in terms of the normative ease (Herkman and Servie, 2013) dimension (means, 3.88 and 3.84; *t* test; *p* = 0.854), with ranges of 2.39–5.29 and 2.42–5.34 in the subsets. This dimension has been shown to be associated with recall probability (Halonen, Kuula, Antila, et al., 2021).

During encoding, 60 written metaphors were shown on a laptop screen, e.g., “Touch is an insect,” for 3,000 ms with a 1,000 ms interval. Participants were instructed to form a mental image of the metaphors. Participants underwent three successive encoding rounds.

In the cued recall, participants were shown the beginning of a metaphor, e.g., “Touch is an”, in a random order and were prompted to type the missing word (e.g., “insect”). Responses were manually scored so that 1 point was given for a correct word and 0.5 points if the response was a synonym (e.g., “bug”) or a higher/lower abstraction (e.g., “mosquito”).

Recall was tested ∼30 min (Day 1), ∼12 h (Day 2), and 4 d (Day 5) after encoding (Fig. 1). The presentation order of pre- and postsleep subsets was counterbalanced. On Day 1 and 2 retrievals, one subset (30 metaphors) was tested, and Day 5 retrieval was administered with all 60 metaphors. Recall outcome was the percentage of correct answers out of all trials. Due to technical problems, one participant's recall data was lost in all retrievals and another participant's data in Day 2 retrieval.

### Self-conscious affect

To induce shame, we used a karaoke-based paradigm. On Day 1 morning (Fig. 1), the participants sang a karaoke version of Abba's Dancing Queen without hearing their own voice through headphones, promoting out-of-tune singing. The experimenter created a ∼1.5 min compilation of the recording without background music using the Audacity 3.0.2 software. The compilation consisted of three selected clips with 5 s silent intervals between the clips.

Both physiological and subjective affective responses to the karaoke playback were measured on Day 1 evening, Day 2 morning, and Day 5 (Fig. 1). Physiological response was assessed with the skin conductance level (SCL). SCL is a measure of tonic electrical conductivity of the skin, and its phasic changes (skin conductance responses, SCRs) reflect autonomic arousal (Braithwaite et al., 2013) and are used experimentally as an accurate marker of stress (Rahma et al., 2022). SCL was measured from the middle and ring fingers of the nondominant hand using a galvanic skin sensor, connected to a Brain Products QuickAmp amplifier (Brain Products). SCL was recorded at a 500 Hz sampling rate and analyzed using MATLAB R2022b (MathWorks). Baseline SCL was obtained from the participant sitting quietly for 5 min; the average baseline measure was calculated after 2 min of quiet sitting, ending 20 s before the playback start (averaged baseline SCL = baseline start +120 s : baseline end −20 s). Next, the participants listened to their singing compilation via a loudspeaker, the experimenter being present. SCL during the playback was averaged. The playback was followed by a 5 min period of quiet sitting. SCR was calculated by dividing playback SCL by baseline SCL and controlling for baseline SCL. Finally, SCR values were square root transformed due to skewedness (Braithwaite et al., 2013).

Subjective embarrassment rating was enquired after each playback: (1) “How ashamed did you feel during the playback?” and (2) “How stressful was it to listen to the playback?”. Rating scale was 0–4 (none–highly). Subjective embarrassment was the mean value of the two ratings and was calculated separately for each playback. No explicit memory tests were administered regarding the karaoke task (singing or playback).

### PSG and sleep fragmentation

All recordings were performed using SOMNOscreen HD (SOMNOmedics) with online monitoring. Gold cup electrodes were attached at six EEG locations (frontal, F3, F4; central, C3, C4; occipital, O1, O2) and at the mastoids (A1, A2). The electrooculogram and the electromyogram were measured using disposable adhesive electrodes (Ambu Neuroline 715, Ambu), two locations for the electrooculogram and three for the electromyogram. An online reference Cz and a ground electrode on the forehead were used. The sampling rate was 256 Hz. PSG data were scored manually using the DOMINO program (v2.9; SOMNOmedics) in 30 s epochs into N1, N2, N3 (SWS), REMS, and wake (American Academy of Sleep Medicine Manual for the Scoring of Sleep and Associated Events). Additionally, in the sleep epochs, we visually identified artifact segments, i.e., pronounced EEG bursts caused by an extracerebral source, e.g., muscle activity and body movements and short arousals (Kane et al., 2017).

To enable comparison between the suppression conditions, we calculated sleep fragmentation. REMS fragmentation was defined as the time spent in either wake, NREMS, or arousals during REMS episodes divided by REMS duration. The first REMS epoch denoted the start of a REMS episode, and the episode ended with the first of at least four continuous minutes of wake or NREMS. SWS episodes and fragmentation were defined otherwise similarly, but SWS episodes concluded with a REMS epoch or 4 min of continuous wake, N1 or N2.

### Sleep spindle detection

The PSG signals were converted to EDF format in the DOMINO software (SOMNOmedics) and then further preprocessed using EEGLAB 14.1.2b (Delorme and Makeig, 2004) running on MATLAB R2018a. Prescored epochs with major (>8 s) artifacts or contact impedance >30 kOhm in the target electrode were excluded from further analyses. All artifact-free signals were digitally bandpass filtered off-line from 0.2 to 35 Hz [Hamming windowed sinc zero-phase finite impulse response (FIR) filter; cutoff, −6 dB], at 0.1 and 35.1 Hz, respectively, and rereferenced to the average signal of the mastoid electrodes.

In this study, we confined our examination to fast sleep spindles due to their documented tendency for SO coupling and their role in memory consolidation (Mölle et al., 2011; Rasch and Born, 2013; Muehlroth et al., 2019; Halonen et al., 2022). Fast sleep spindles were detected in central electrodes according to their topological distribution (De Gennaro and Ferrara, 2003). We used individual frequency bands for spindle detection. First, for each participant, we detrended their power spectral density (PSD) curve and extracted the PSD peak frequency within the sigma range (9–16 Hz). In case two sigma peaks were visually observed, we selected the one with a higher frequency. The sample's mean peak frequency for fast spindle detection was 13.47 (SD, 0.56) Hz. Next, we detected sleep spindles with an automated algorithm adapted from Ferrarelli et al. (2007) using the Wonambi EEG analysis toolbox (Piantoni and O’Byrne, 2023; https://wonambi-python.github.io/). EEG data were bandpass filtered with a zero-phase equiripple Chebyshev FIR filter in individual mean peak frequency ±2 Hz. The threshold values for finding the channel-wise spindle peak amplitude were defined by multiplying the mean channel amplitude (µV) by 5. The putative spindle's amplitude was required to stay over the mean channel amplitude multiplied by 2 for 250 ms in both directions from the peak. The maximum cutoff for spindle length was 3.0 s. We ran the detection separately for N2 and SWS epochs and excluded spindle-like bursts that occurred during artifacts. Fast spindle density was calculated by dividing the total spindle number by minutes spent in N2/SWS.

### SO detection

SOs were detected in central electrodes with an algorithm adapted from Ngo et al. (2015) using the Wonambi toolbox (Piantoni and O’Byrne, 2023). The EEG signal was first low-pass filtered at 3.5 Hz. All negative and positive amplitude peaks were identified between consecutive positive-to-negative zero-crossings. Zero-crossing intervals within 0.8–5 s (0.2–1.25 Hz) were included. Mean values for positive and negative peak potentials were calculated, and events were denoted as SOs where the negative peak was lower than the mean negative peak multiplied by 0.9 and where the positive-to-negative peak amplitude difference exceeded the mean amplitude difference multiplied by 0.9. We ran the detection separately on N2 and SWS epochs.

### SO–spindle coupling

**Figure 2.**
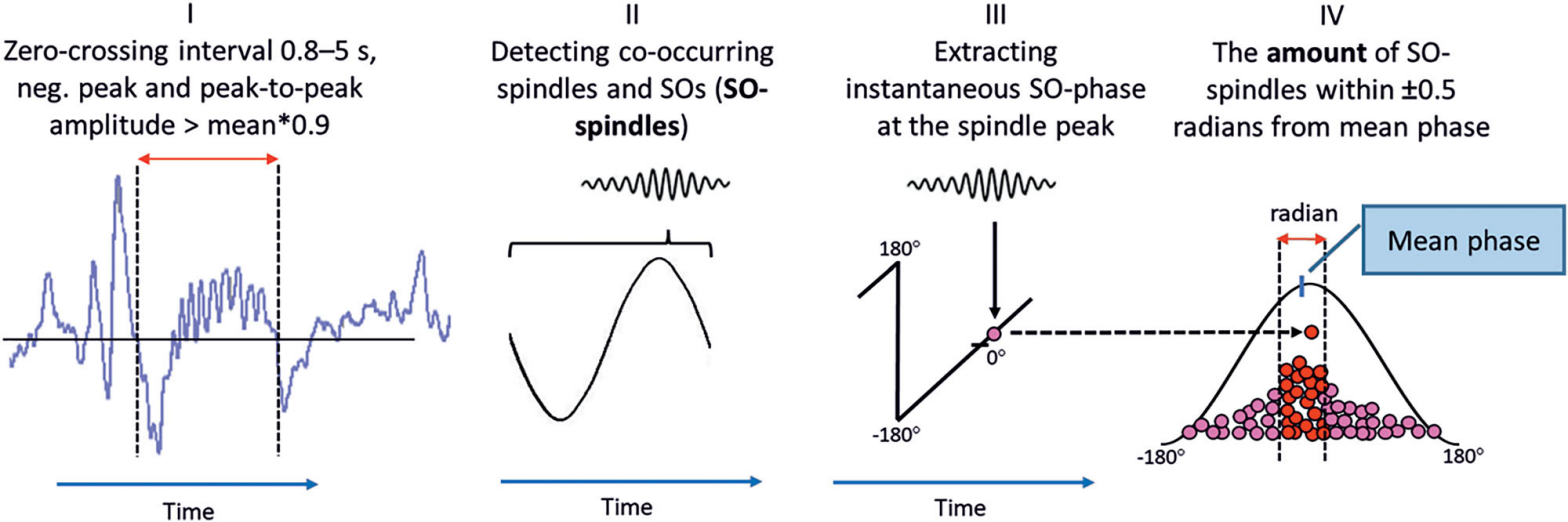
Quantifying SO–spindle coupling. ***I***, Detecting SOs. ***II***, Detecting spindles that peak within the SO cycle. ***III***, After Hilbert transformation, extracting the SO phase at the instant of the spindle peak. ***IV***, Averaging the phase of all SO–spindles and calculating the number of spindles that peak within ±0.5 radian from the mean phase.

We identified fast spindles where the amplitude peaked within a SO cycle (SO-coupled spindles). Next, we bandpass filtered the EEG signal to 0.2–1.25 Hz, and after Hilbert transformation, extracted the instantaneous phase at the SO-coupled spindle peaks. Channel-wise resultant vector length and circular mean phase were calculated using Python implementation of the CircStat toolbox (Berens, 2009; https://github.com/circstat/pycircstat). Finally, we calculated the number of SO-coupled spindles that peaked within ±0.5 radians from the individual mean phase (optimally coupled spindles, OC-spindles). Resultant vector length and OC-spindle values between central electrodes were averaged. The procedure was conducted separately on N2 and SWS epochs (Figure 2).

### Power spectral density

PSD was calculated for N2, REMS, and SWS epochs in frontal channels using “spectopo” function of EEGLAB with a window length of 1,024 samples (4 ms) and an overlap of 50% resulting in bins with 0.25 Hz resolution. We averaged the PSD values into 1 Hz bins between 0 and 30 Hz and further between F4 and F3 electrodes. Next, we *z*-scored the stage-specific 1 Hz PSD bin values across all bins and participants and linearly transferred them into positive range (minimum value of 1) and summed the accumulated power across all REM/SWS epochs (PSD energy). The REMS/SWS bin-wise sum scores were controlled for mean PSD (across 0–30 Hz) of which the effects of the suppression condition and sleep stage duration were partialled out. This was done in order to control for amplitude variation due to variable conductance (e.g., skull and skin properties). Finally, we averaged the 1 Hz bins to SO delta (≤ 4 Hz), theta (4–8 Hz), alpha (8–12 Hz), sigma (12–16 Hz), beta1 (16–22 Hz), and beta2 (22–30 Hz) ranges.

### Questionnaires

Prior to participation, the participants filled in questionnaires for shame-proneness (Test of Self-Conscious Affect-3, Finnish version; Tangney et al., 2000), depression symptoms (Beck Depression Inventory II; Beck et al., 1996), and anxiety symptoms (Generalized Anxiety Disorder 7; Williams, 2014). Additionally, the highest achieved education level was queried (1, primary/lower secondary education; 2, upper secondary education; 3, bachlor's degree; 4, master's degree).

Participants were screened for their experience in performing and singing with the questions “Previous performing experience, e.g., speeches, presentations, acting, or singing?” and “Do you sing at leisure time or work?” on a scale of 1–5 (none–very much). The mean score was denoted as “singing experience.”

### Statistical power

No a priori power analyses were conducted. However, the sample size (*N* = 29) paralleled experimental studies applying mixed (Rosales-Lagarde et al., 2012; Spoormaker et al., 2012; Halonen, Kuula, Makkonen, et al., 2021) or within-subject designs (Werner et al., 2015). Sensitivity analysis with G*Power 3.1.9.2 (Faul et al., 2007) showed a minimum detectable effect size of *f* = 0.43 (large; calculated “as in SPSS”) for interaction and within-factors in a 2 (between) × 3 (within) mixed ANOVA (*N* = 29; two-tailed *α* = 0.05; power = 0.8). For between-factors, the minimum detectable effect size of *f *= 0.56 (bordering very large), suggesting an increased possibility for false-negatives (Type 2 error). For bivariate correlations in one sample (*N* = 29), the medium–large effect size of *r* ≥ 0.44 is required (two-tailed *α *= 0.05; power = 0.8).

### Statistics

Data were analyzed using IBM SPSS Statistics for Windows, version 29.0 (IBM), or R, version 4.3.2.

The distributions of the dependent and independent variables were investigated for violations of normality (visually and with Shapiro–Wilk test) and Hartigan's dip test for unimodality (Hartigan and Hartigan, 1985) due to our dichotomous suppression paradigm. Residual scatterplots were visually investigated for heteroscedasticity. The equality of variances between the conditions in all dependent variables was tested with Levene's test. Observations were considered outliers if they deviated at least 2.5 SD from the sample mean in its static variable (e.g., SCR on Day 1) and in its change score (e.g., SCR change between Days 1 and 2) or fitted versus residual value.

In preliminary analyses, we examined whether emotional response (SCR and subjective) and memory scores are associated with age and questionnaire scores (Pearson's correlation) or differed between sexes (one-way ANOVA). The associations between baseline night sleep duration and Day 1 evening measurements (baseline SCL, SCR, subjective embarrassment, and metaphor recall) were tested with Pearson's correlation. χ^2^ was used to test sex distribution between the conditions (REMS_SUPPR_ / SWS_SUPPR_). Condition differences in age, questionnaire scores, sleep, and oscillatory variables were tested with one-way ANOVA.

We used linear mixed model (LMM; restricted maximum likelihood and random ID intercept) for the main analyses regarding the effects of suppression condition and sleep parameters on emotional response and memory retrieval. LMMs are robust against slight violations of distributional assumptions (Schielzeth et al., 2020) and avoid listwise deletion when a value is missing/excluded.

Baseline versus playback SCL difference in the three measurements (Day 1, Day 2, and Day 5) was tested with a “2 × 3” structure LMM. LMM was used to test the main effect of time (repeated variable, three levels) and suppression condition (between-subjects, two levels) and their interaction (“time × condition”) on SCR, subjective embarrassment, and metaphor recall. LMM was also used to examine the main effects and time interactions of PSD energy values during REMS and SWS on both SCR and subjective embarrassment, as well as the main effects and time interactions of spindle/SO–spindle variables on metaphor recall. We ensured LMM covariance structure fit with corrected Akaike's information criterion (Hurvich and Tsai, 1989). Regarding LMMs on emotional response, “Factor analytic: First order” showed the best fit. In metaphor recall “Compound Symmetry: Heterogeneous” was applied.

Significant LMM effects were followed up with repeated-measure *t* tests (change between two measurements) or linear regressions (condition differences and slope between change score and independent variable). The associations between overnight SCR change and PSD energy 1 Hz bins were explored with Pearson's correlation.

All analyses on subjective embarrassment and metaphor recall were controlled for sex. LMMs testing the “time × condition” interaction on SCR were first run unadjusted and rerun using the number of awakenings as a covariate. Regarding subjective embarrassment, we reran the “time × condition” interaction with baseline night sleep duration as a covariate.

The nominal level of statistical significance was set at *α *= 0.05. The preliminary analyses and condition comparisons in sample characteristics were run without multiple-test correction. Bonferroni’s correction was applied on LMMs with PSD energy (across six frequency bands) and on LMMs with spindle density and OC-spindle number (two tests). Follow-up tests were Bonferroni-corrected (repeated measures in the whole sample/within condition across three/six tests; regressions on static values/change scores across three/two tests).

As supplementary information, nonparametric equivalents for the follow-up analyses were run including the outliers: Friedmann's test for repeated-measure testing in the whole sample and within-condition, Mann–Whitney *U* test for between-condition comparisons in emotional responses and their change, and Spearman's correlation for the associations between PSD values and emotional response change. Bonferroni’s corrections were applied as in the parametric follow-up tests, but PSD band associations were corrected over six tests.

## Results

### Sample characteristics and condition

Total sleep times were similar between the suppression conditions, but REMS_SUPPR_ had 48% less REMS than SWS_SUPPR_ (*p* < 0.001), and SWS_SUPPR_ had 50% less SWS than REMS_SUPPR_ (*p* < 0.001). Significant sleep/oscillatory differences between the conditions were found in N2 duration, REMS/SWS fragmentation, SO-coupled spindle number in SWS, and PSD energy values in REMS and SWS. Questionnaire scores or highest achieved education did not differ between the conditions, but singing experience and the number of forced awakenings were higher in the SWS_SUPPR_ condition (Table 1).

**Table 1.** Sample characteristics and sleep parameters

	Mean (SD)		*p*
	REMS_SUPPR_ (*n* = 15)	SWS_SUPPR_ (*n* = 14)	
Age (*y*)	26.3 (4.4)	25.3 (4.8)	0.571
BDI-II	6.0 (5.7)	6.4 (6.5)	0.877
GAD-7	3.6 (3.4)	3.5 (3.3)	0.936
TOSCA 3 Shame	42.3 (11.4)	41.6 (13.2)	0.892
Education	2.3 (0.8)	2.6 (0.9)	0.348
Singing experience	2.3 (0.9)	3.1 (0.9)	0.015*
Sleep characteristics			
TST baseline night	7:05 (0:38)	7:13 (0:31)	0.539
TST experimental	6:04 (0:27)	6:10 (0:19)	0.548
N1	0:42 (0:16)	0:32 (0:12)	0.052
N2	3:03 (0:30)	3:27 (0:30)	0.039*
SWS	1:36 (0:29)	0:48 (0:21)	<0.001***
REMS	0:43 (0:19)	1:23 (0:18)	<0.001***
WASO	0:53 (0:21)	0:49 (0:20)	0.548
REMS Fragmentation %	42.4 (21.3)	7.1 (8.1)	<0.001***
SWS fragmentation %	13.5 (9.0)	39.2 (17.3)	<0.001***
Forced awakenings, *N*	10.1 (3.3)	12.7 (2.9)	0.031*
Awakening strength	1.9 (0.2)	1.6 (0.3)	0.002**
REMS power spectral density energy			
SO delta	122.2 (42.5)	206.2 (35.2)	<0.001***
Theta	104.5 (56.4)	219.4 (49.1)	<0.001***
Alpha	109.3 (54.7)	227.8 (51.9)	<0.001***
Sigma	143.1 (62.9)	247.4 (49.0)	<0.001***
Beta 1	125.3 (48.9)	292.7 (54.0)	<0.001***
Beta 2	125.3 (37.5)	252.4 (71.5)	<0.001***
SWS power spectral density energy			
SO delta	311.8 (161.2)	157.8 (92.4)	0.004**
Theta	275.6 (133.6)	160.8 (88.1)	0.012*
Alpha	277.7 (193.1)	165.2 (91.9)	0.058
Sigma	353.4 (183.4)	207.8 (102.1)	0.014*
Beta 1	287.1 (131.9)	182.2 (84.8)	0.018**
Beta 2	307.1 (124.7)	192.3 (87.5)	0.008**
SWS SO–spindle			
Fast spindle *N*	373.5 (142.7)	190.5 (115.7)	<0.001***
Fast spindle density	4.0 (1.6)	4.2 (1.7)	0.762
SO–spindle *N*	82.4 (32.7)	39.1 (25.0)	<0.001***
Resultant vector length	0.53 (0.10)	0.55 (0.19)	0.709
N2 SO–spindle			
Fast spindle *N*	927.1 (341.1)	1,037.9 (312.2)	0.371
Fast spindle density	5.0 (1.2)	5.0 (1.1)	0.947
SO–spindle *N*	137.2 (41.9)	149.9 (48.9)	0.458
Resultant vector length	0.37 (0.17)	0.41 (0.20)	0.564

BDI, Beck Depression Inventory; GAD-7, Generalized Anxiety Disorder 7 questionnaire; TOSCA, Test of Self-Conscious Affect; TST, total sleep time; SWS, slow-wave sleep; N1-2, sleep stage 1–2; REMS, rapid eye movement sleep; WASO, wake after sleep onset; SO, slow oscillation.

*** <0.001.

** <0.01.

* <0.05.

Examining the distributions revealed extreme outliers: in one participant, both Day 1 SCR and overnight SCR change exceeded the sample mean by 3.4 SD and 3.0 SD, respectively. Another participant showed +2.8 SD and +3.0 SD in Day 2 SCR and overnight SCR change, respectively. In this participant, SWS PSD energy values exceeded the sample mean by 3.2 SD on average (2.6–3.7 SD). We excluded these outlying SCR and PSD values from LMM and follow-up analyses. Extended Data Figure 3-1*A–D* shows the excluded variables. Subjective embarrassment at Day 5 was not normally distributed (Shapiro–Wilk *p* = 0.016), and the Day 2 to Day 5 change was not normally (*p* < 0.001) or unimodally (Hartigan's test *p* < 0.008) distributed.

Preliminary analyses showed no significant associations between background variables (age, questionnaire scores) and SCR/memory scores (all *p* ≥ 0.05). Females had a significantly higher metaphor recall percentage on Day 1 (68.8% vs 53.0%), Day 2 (68.1% vs 47.1%), and Day 5 (65.5% vs 41.5%) retrievals (all *p* ≤ 0.046) than males, as well as higher subjective embarrassment ratings on Day 2 (1.37 vs 0.64; *p* = 0.021) and Day 5 (1.13 vs 0.46; *p* = 0.029). Sleep duration on the baseline night correlated significantly (negatively) with subjective embarrassment (*r* = −0.373; *p* = 0.046), but not other evening measures (*p* ≥ 0.190).

### Stress induction and SCR

**Figure 3.**
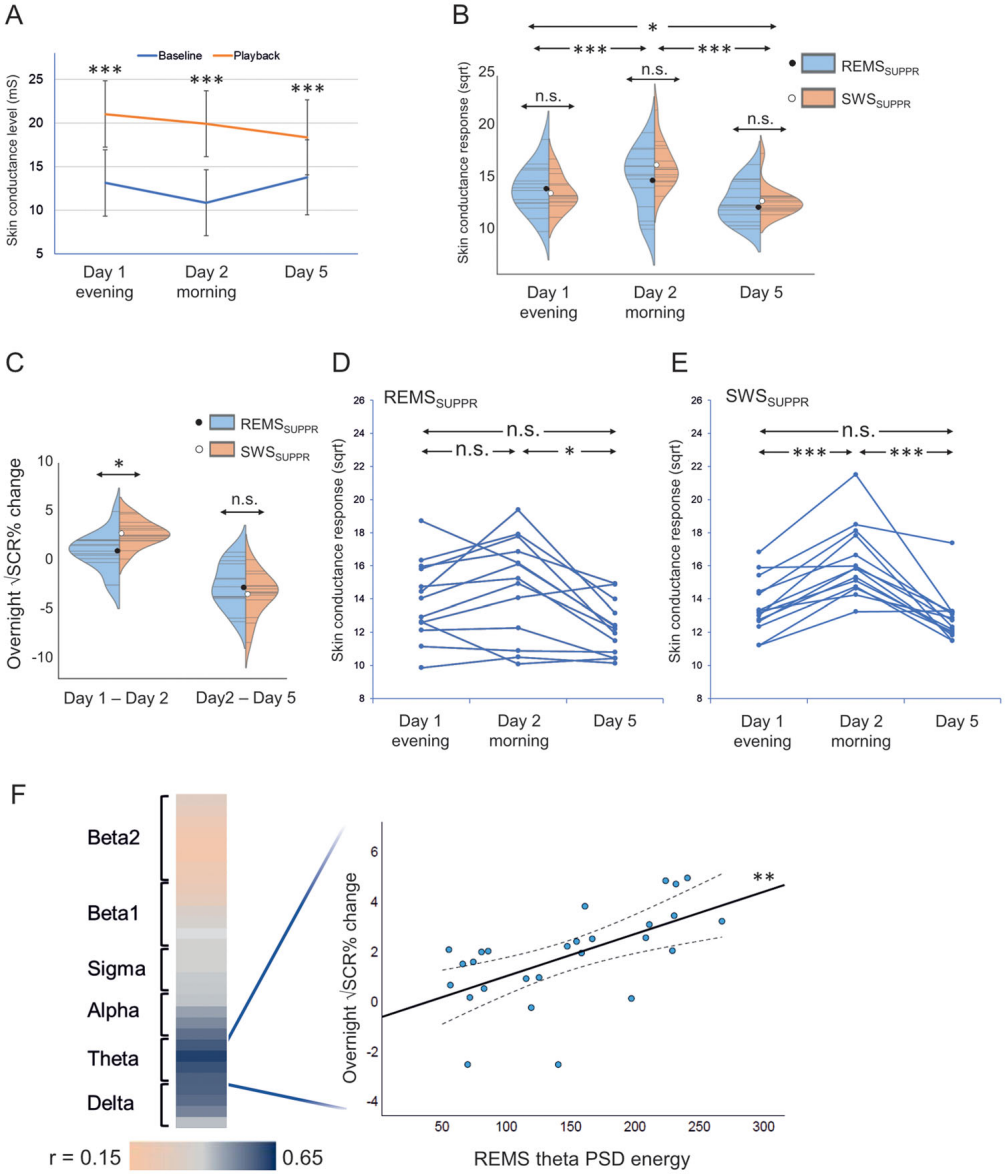
Skin conductance, suppression condition, and REMS oscillations. The playback of the karaoke recording induced a significant rise in SCL in all measurements (*p* < 0.001, Bonferroni-corrected; ***A***). Violin plots illustrate individual (gray lines) and condition-wise (blue/orange) distributed SCRs with mean values (black/white dot) across the three measurements (***B***). Overnight change in SCR was higher in SWS_SUPPR_ than in REMS_SUPPR_ (*p* = 0.016, Bonferroni-corrected; ***C***). Individual trajectories of SCRs in REMS_SUPPR_ (***D***) and in SWS_SUPPR_ (***E***). The scatterplot illustrates the positive association between REMS theta power spectral density (PSD) energy and overnight SCR change (*p* = 0.002, Bonferroni-corrected), and the heatmap displays Pearson’s correlation strength across 0–30 Hz for REMS (***F***). Error bars and dashed lines: 95% confidence interval. ***p < 0.001; **p < 0.01; *p < 0.05; Bonferroni-corrected. Extended Data Figure 3-1 displays the excluded outliers.

SCL was significantly higher during the playback than the baseline (*F*_(2,27.676)_ = 61.301; *p* < 0.001) in all measurements (post hoc *p* < 0.001; Cohen's *d* ≥ 1.333; Fig. 3*A*). Regarding SCR, we found a significant time effect (*F*_(2,24.425)_ = 21.906; *p* < 0.001), indicating that SCR varied between Days 1, 2, and 5 measurements. Bonferroni-corrected follow–up tests showed that SCR was higher on Day 2 (*M* = 15.54; SD = 2.62) than on Day 1 (*M* = 13.75; SD = 1.98) and Day 5 (*M* = 12.73; SD = 1.69; *t*_(26)_ = 4.973, *p* < 0.001, Cohen's *d* = 0.96 and *t*_(27)_ = 6.573, *p* < 0.001, Cohen's *d* = 1.35, respectively) and on Day 1 than Day 5 (*t*_(27)_ = 3.170; *p* = 0.011; Cohen's *d* = 0.60; Fig. 3*B*). No significant difference was found in overall (across all measurements) SCR between REMS_SUPPR_ and SWS_SUPPR_ conditions (main effect *F*_(1,25.952)_ = 0.091; *p* = 0.766).

10.1523/ENEURO.0453-23.2024.f3-1 Figure 3-1 Download Figure 3-1, TIF file.

The “condition × time” interaction was significant (*F*_(2,24.332)_ = 4.209; *p* = 0.027). Controlling for the amount of awakenings did not change the significance status of “condition × time” (*F*_(2,23.576)_ = 4.284; *p* = 0.026). Following up the interaction by comparing the SCR change between the conditions showed that the overnight SCR change (Day 1 to Day 2) was higher in SWS_SUPPR_ (*M* = 2.69; SD = 1.30; percentual increase, *M* = 20.1%; SD = 9.3%) than in REMS_SUPPR_ (*M* = 0.84; SD = 1.97; *M* = 6.1%; SD = 13.6%; *t*_(1,25)_ = 2.906; *p* = 0.016; *R*^2 ^= 0.253), but the conditions did not differ in the Day 2 to Day 5 SCR change (REMS_SUPPR_, *M* = −2.28; SD = 2.38; *M* = −15.9%; SD = 10.8%; SWS_SUPPR_, *M* = −3.47; SD = 2.18; *M* = −20.5%; SD = 10.7%; *t*_(26) _= −1.373; *p* = 0.181; *R*^2 ^= 0.068; Fig. 3*C*).

Within-condition repeated–measure tests showed that in REMS_SUPPR_, the SCR was significantly higher on Day 2 (*M* = 14.82; SD = 2.92) than on Day 5 (*M* = 12.68; SD = 1.92; *t*_(13)_ = 3.600; *p* = 0.019; Cohen's *d* = 0.962). Day 1 SCR (*M* = 13.94; SD = 2.31) did not significantly differ from Day 2 (*t*_(12) _= −1.530; *p* = 0.912; Cohen's *d* = 0.424) or Day 5 measurements (*t*_(13)_ = 2.673; *p* = 0.106; Cohen's *d* = 0.714; Fig. 3*D*). In SWS_SUPPR_, Day 2 SCR (*M* = 16.26; SD = 2.14) was significantly higher than Day 1 (*M* = 13.57, SD = 1.65; *t*_(13)_ = 7.752; *p* < 0.001; Cohen's *d* = 2.072) and Day 5 SCRs (*M* = 12.79; SD = 1.46; *t*_(13)_ = 5.958; *p* < 0.001; Cohen's *d* = 1.592). No significant difference was found between Day 1 and Day 5 measurements (*t*_(13)_ = 1.735; *p* = 0.638; Cohen's *d* = 0.464; Fig. 3*D*). Between the conditions, SCR did not differ significantly on Day 1 (*t*_(1,26) _= −0.485; *p* = 1.000; *R*^2 ^= 0.009), Day 2 (*t*_(1,26)_ = 1.484; *p* = 0.450; *R*^2 ^= 0.078), or Day 5 (*t*_(1,27)_ = 0.170; *p* = 0.866; *R*^2 ^= 0.001; Fig. 3*E*).

In sum, selective sleep stage suppression elicited pronounced overnight SCR increase in the condition with suppressed SWS (intact REMS) compared with that in REMS-suppressed condition.

### SCR and power spectral density

PSD energy in any frequency band was not significantly associated with overall SCR in REMS or SWS (Table 2). In REMS, significant “time × PSD” interaction on SCR was found regarding theta band (*F*_(2,26.166)_ = 7.002; *p* = 0.023), indicating that theta PSD energy is associated with SCR change between measurements. No other significant time interactions were found regarding PSD energy in REMS or SWS (all *p* ≥ 0.165, Bonferroni-corrected; Table 2).

**Table 2.** LMM results: emotional response by condition and power spectral density energy values

	SCR				Subjective embarrassment			
	Main effect		*X* time		Main effect		*X* time	
	*F*	*p*	*F*	*p*	*F*	*p*	*F*	*p*
Condition	0.091	0.766	4.209	0.027*	0.216	0.646	2.056	0.148
REMS PSD								
SO delta	0.010	1.000	4.191	0.165	0.080	1.000	2.548	0.581
Theta	0.019	1.000	7.002	0.023*	0.069	1.000	3.658	0.236
Alpha	0.833	1.000	3.325	0.314	0.249	1.000	2.002	0.927
Sigma	0.316	1.000	1.575	1.000	0.252	1.000	1.635	1.000
Beta1	0.152	1.000	1.062	1.000	0.219	1.000	2.518	0.596
Beta2	0.115	1.000	0.597	1.000	0.299	1.000	2.412	0.652
SWS PSD								
SO delta	0.017	1.000	0.135	1.000	2.025	1.000	1.589	1.000
Theta	0.004	1.000	0.065	1.000	1.673	1.000	1.878	1.000
Alpha	0.000	1.000	0.217	1.000	1.020	1.000	1.491	1.000
Sigma	0.008	1.000	0.115	1.000	2.266	0.869	2.818	0.468
Beta1	0.005	1.000	0.325	1.000	2.698	0.678	3.872	0.202
Beta2	0.065	1.000	0.397	1.000	2.992	0.576	4.840	0.098

*X* time, interaction between the examined variable and time (three measurements); REMS, rapid eye movement sleep; SWS, slow-wave sleep; PSD, power spectral density. REMS/SWS PSD *p* values Bonferroni-corrected across six tests.

* *p* < 0.05. See Extended Data Table 2-1 for nonparametric tests including outliers.

10.1523/ENEURO.0453-23.2024.t2-1 Table 2-1 The effects of suppression condition and power spectral density values on emotional response values/change in non-parametric statistics. Download Table 2-1, DOC file.

Follow-up examination on the significant “time × REMS theta PSD” interaction showed a robust positive association between REMS theta PSD energy and overnight SCR change (*t*_(1,25)_ = 3.762; *p* = 0.002; *R*^2 ^= 0.336) but not Day 2 to Day 5 SCR change (*t*_(1,26) _= −1.475; *p* = 0.304; *R*^2 ^= 0.077). Figure 3*F* displays the scatterplot between REMS theta PSD energy and overnight SCR change and heatmapped correlation strengths between bin-wise (0–30 Hz) PSD energy and overnight SCR change. See Extended Data Figure 3-1*E* for the REMS theta–overnight SCR change including the excluded outliers.

### Subjectively rated embarrassment

**Figure 4.**
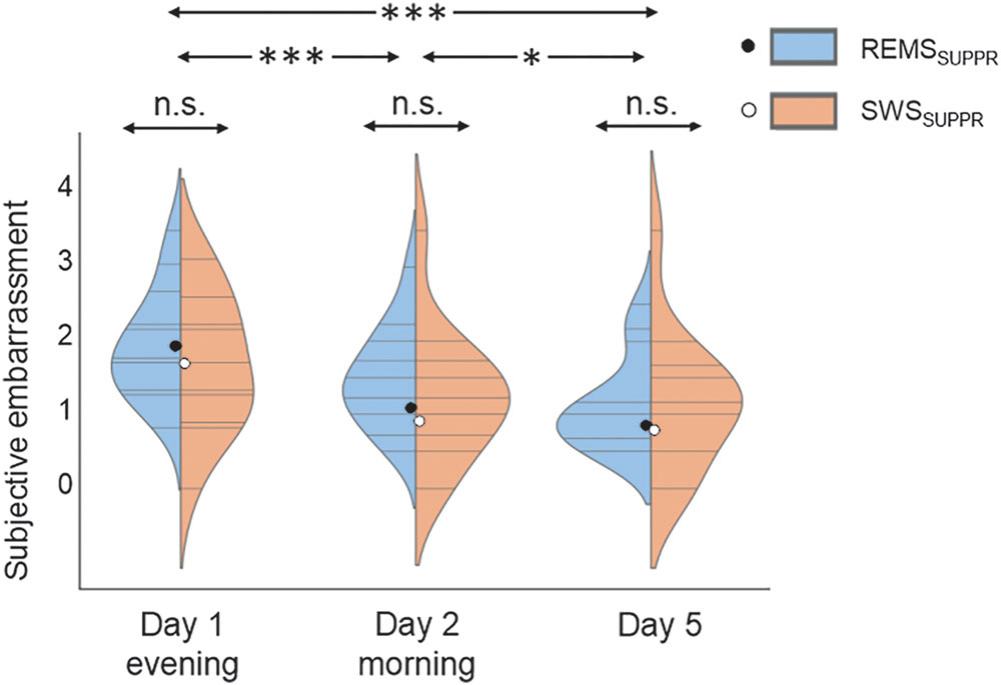
Subjective embarrassment. A violin plot illustrating individual (gray lines) and condition-wise (blue/orange) distributed embarrassment ratings with mean values (black/white dot) across the three measurements.

Subjectively rated embarrassment differed according to time (*F*_(2,28.029)_ = 27.058; *p* < 0.001). Follow-up repeated–measure tests showed that subjectively rated embarrassment was higher on Day 1 (*M* = 1.83; SD = 0.93) than on Day 2 (*M* = 1.02; SD = 0.86; *t*_(28)_ = 7.097; *p* < 0.001; Cohen's *d* = 1.32) and Day 5 (*M* = 0.81, SD = 0.82) [*t*_(28)_ = 7.089, *p* < 0.001, Cohen's *d* = 1.32]. Additionally, embarrassment on Day 2 was rated higher than on Day 5 (*t*_(28)_ = 2.820; *p* = 0.027; Cohen's *d* = 0.52; Fig. 4). Suppression conditions did not differ in overall (across all measurements) subjective embarrassment between the suppression conditions (*F*_(1,26.000) _= 0.216; *p* = 0.646). “Condition × time” interaction was not significant unadjusted (*F*_(2,27.024)_ = 2.056; *p* = 0.148) or after controlling for baseline night sleep duration (*F*_(2,27.011)_ = 1.252; *p* = 0.302). In sum, subjectively rated embarrassment decreased in all measurements, while suppression condition did not induce significant differences.

Regarding REMS/SWS PSD energy, none of the frequency bands showed a significant main effect on subjective embarrassment (*p* ≥ 0.576). The “time × PSD energy” interaction was not significant in either REMS (*p* ≥ 0.236) or SWS (*p* ≥ 0.098) in any frequency band. See Table 2 for full results. We explored how excluding the marginally nonoutlier in SWS PSD (Extended Data Figure 3-1*D*, second highest dot) affected the LMMs regarding PSD and emotional responsivity. No changes in significance status emerged (all *p* ≥ 0.429, Bonferroni-corrected; data not shown).

### Metaphor recall and NREMS oscillations

**Figure 5.**
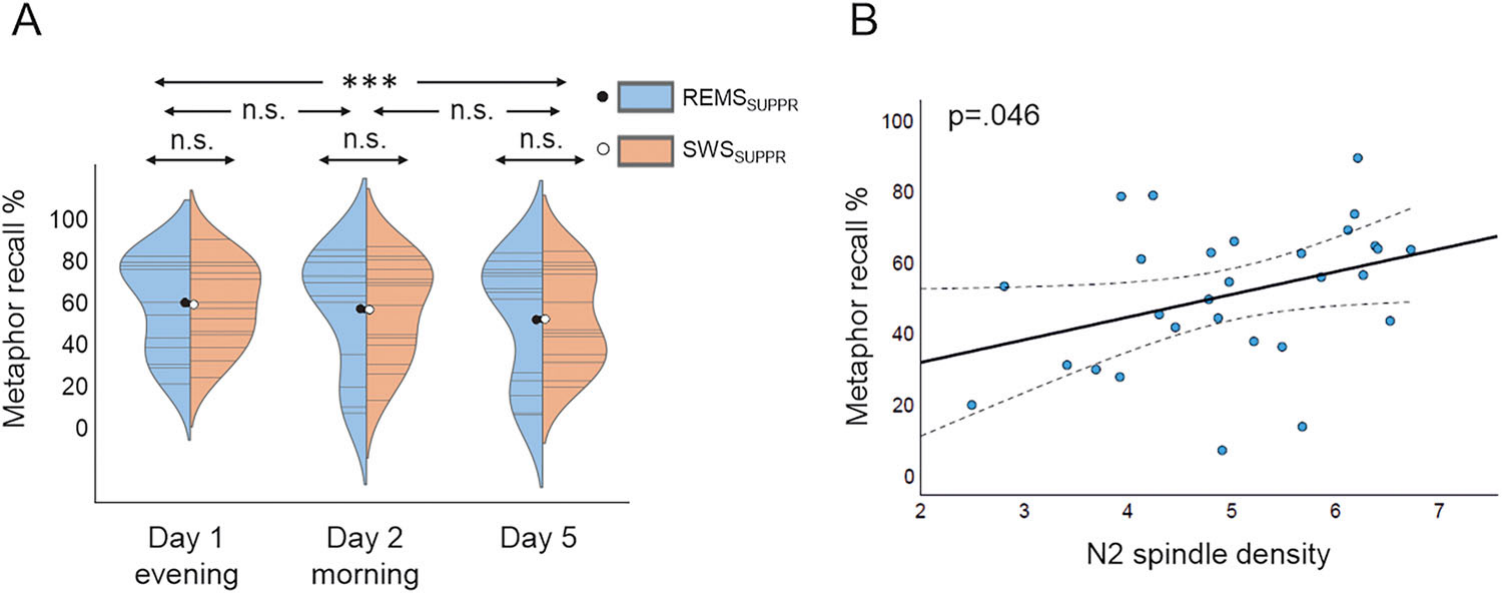
Metaphor recall. A violin plot illustrates individual (gray lines) and condition-wise (blue/orange) distributed metaphor recall percentages with mean values (black/white dot) across the three measurements (***A***). N2 fast spindle density predicted positively overall metaphor recall (averaged across all retrievals; *p* = 0.046, Bonferroni-corrected) (***B***). Dashed lines: 95% confidence interval.

Time had a significant main effect on metaphor recall (*F*_(2,26.671)_ = 12.508; *p* < 0.001). Follow-up paired-sample *t* tests showed that the Day 1 recall percentage (*M* = 61.4%; SD = 21.1%) was significantly higher than that of Day 5 (*M* = 54.4%; SD = 25.7%; *t*_(27)_ = 4.817; *p* < 0.001; Cohen's *d* = 0.910) but not on Day 2 (*M* = 58.8%; SD = 25.4%; *t*_(26)_ = 1.433; *p* = 0.492; Cohen's *d* = 0.276). The difference between Day 2 and Day 5 was not statistically significant (*t*_(26)_ = 1.371; *p* = 0.546; Cohen's *d* = 0.264). The condition did not have a significant main effect (*F*_(1,24.978)_ = 0.056; *p* = 0.816) nor time interaction (*F*_(2,25.666)_ = 0.456; *p* = 0.639) on the metaphor recall percentage (Figure 5*A*).

Fast spindle density or OC-spindle number in SWS did not associate significantly with the overall recall percentage (*F*_(1,25.003)_ = 4.630, *p* = 0.081 and *F*_(1,24.975)_ = 2.757, *p* = 0.219, respectively). Their interaction with time was also not significant (*F*_(2,25.828)_ = 0.659, *p* = 1.000 and *F*_(2,25.752)_ = 1.386, *p* = 0.536, respectively). The main effect of fast spindle density in N2 sleep was significant (*F*_(1,25.012)_ = 5.876; *p* = 0.046), i.e., N2 spindle density associated positively with the overall recall percentage (Fig. 5*B*), but its time interaction was not (*F*_(2,25.803)_ = 0.719; *p* = 0.994). No significant findings were observed regarding N2 OC-spindles (main effect, *F*_(1,24.989)_ = 5.583; *p* = 0.052; time interaction, *F*_(2,25.747)_ = 0.867; *p* = 0.864).

Thus, suppression condition, or its impact on accumulated SO–spindle events, did not associate with significant differences in metaphor recall.

## Discussion

To better understand the role of REMS and SWS in off-line affective and declarative memory processing, we applied overnight selective sleep stage suppression. As assumed, the two sleep suppression conditions resulted in different outcomes regarding the modulation of emotional response from the pre- to postsleep period. A greater pre- to postsleep increase in physiological reactivity to an emotional stressor was observed after suppressed SWS (intact REMS), relative to suppressed REMS (intact SWS), providing evidence that affective processing is sleep stage-dependent. The overnight increase in emotional responsivity toward the stressor was positively associated with REMS theta activity. The retention of neutral declarative material did not differ between the suppression conditions and was not associated with (SO–)spindle parameters.

The physiological response toward the emotional stressor increased significantly between evening- to-morning measurements in the condition with suppressed SWS, i.e., intact REMS. We interpret this from the perspective of REMS quality, as the overnight SCR change was strongly associated with the accumulated REMS theta (4–8 Hz) energy, beyond any other oscillatory band. Indeed, REMS and theta energy seemed to preserve (or even strengthen) the affective component of the stress memory. This aligns with studies linking the amount of REMS to elevated/preserved subjective or physiological postsleep emotional response (Lara-Carrasco et al., 2009; Pace-Schott et al., 2011, 2015; Baran et al., 2012; Gilson et al., 2015; Werner et al., 2015, 2021; Halonen, Kuula, Makkonen, et al., 2021). The significance of REMS theta oscillations in off-line affective memory consolidation is supported by animal models and studies on emotional learning in humans (Nishida et al., 2009; Popa et al., 2010; Prehn-Kristensen et al., 2013; Sopp et al., 2017; Kim et al., 2020). During REMS, theta generation is tied to pontine–geniculo–occipital waves (Hutchison and Rathore, 2015), which instantiate limbic activity and facilitate emotional memory processing (Mavanji et al., 2004; Datta et al., 2008; Karashima et al., 2010).

However, the effect of REMS on affective memories is not univocal in the previous literature. Our results contradict the forgetting part of the SRSF hypothesis, which argues that especially REMS serves to detune the affective charge from recent memories (Walker and van der Helm, 2009). In support of the SRSF hypothesis, several studies associate the amount of REMS with lower postsleep affective response (Gujar et al., 2011; Rosales-Lagarde et al., 2012; Spoormaker et al., 2012; Wassing et al., 2019). The discrepancy appears to not be related to the type of stress assessment (i.e., subjective or physiological). For example, SCR toward conditioned fear of electric shocks in the study by Spoormaker et al. (2012) was detuned via REMS, whereas another study (Halonen, Kuula, Makkonen, et al., 2021) reported a positive association between REMS and karaoke playback-induced SCR. Importantly, these apparently opposite findings urge one to consider what is being consolidated by REMS. In the earlier karaoke study (Halonen, Kuula, Makkonen, et al., 2021), and in the present study, REMS proposedly strengthened the memory of social stress. On the other hand, Spoormaker et al. (2012) administered a presleep extinction task for a previously formed fear memory. The next day, cueing the fear memory elicited elevated SCR in those whose REM sleep was deprived, relative to intact sleepers. Thus, REMS may either detune (Spoormaker et al., 2012) or strengthen the physiological emotional response, as evidenced by conditioned fear of electric shocks (Menz et al., 2013) and social stress (Halonen, Kuula, Makkonen, et al., 2021). This bidirectionality may adaptively depend on the presleep conditions.

From an evolutionary perspective, fully and rapidly downscaling the charge of all emotional experiences may not be favorable—affective charge ensures the readiness to respond rapidly to threat in natural environments. Sleep may then have an adaptive function in the regulation of affect, such as fear. However, human emotional landscape is more complicated. To put the study in a related context, we applied the concept of social stress linked to shame and social self-representation, equally assumed to have a social evolutionary origins (Tracy and Robins, 2004) and to be a factor in mood disorders like depression (Kim et al., 2011). Indeed, threats to the social self have been shown to induce physiological stress responses, as evidenced in studies applying the Trier Social Stress Test (Ruiz et al., 2010) and the out-of-tune singing paradigm as in the current study (Wassing et al., 2019; Halonen, Kuula, Makkonen, et al., 2021). Shame, in the short term, may motivate “repairing” actions in social contexts (Xing et al., 2021), thus serving adaptivity. In our study, the effect of REMS in preserving the physiological response was short-lived, as no evident sleep effects remained after a few days. Likely, the intervening time and normalized sleep attenuated any excess response to it.

Subjective embarrassment decreased substantially between the evening and morning playbacks. While this would point to sleep-related habituation of subjective stress, the interpretation may be more complex. First, neither the suppression condition (REMS/SWS) nor sleep PSD measures influenced the change in subjective embarrassment from pre- to postsleep measurements. The finding contradicts the SRSF hypothesis claiming that specifically REMS and theta would attenuate the affective response toward the stressor (Walker and van der Helm, 2009). One possibility is that not only REMS, but also SWS, contributed to the regulation of the subjective affect. Evidence of this mechanism is provided by Talamini et al. (2013), showing how overnight emotional habituation to subjectively rated emotional distress was associated with a compensatory response and trait characteristics of SWS. An alternative explanation is that merely repeating the stressor was the main cause of the attenuation. In a previous study using a similar stressor, subjective embarrassment ratings decreased pronouncedly between the first and second playbacks, during a period spent awake (Halonen, Kuula, Makkonen, et al., 2021). Habituation by repetition may have overshadowed any sleep (stage)-specific effects in the present study. Finally, subjective postsleep ratings verged on a floor effect, and especially the measurements (and change) regarding Day 5 embarrassment were compromised by nonnormal and nonunimodal distribution. These statistical properties, along with a small sample's (*N* = 29) inclination to underdetect sublarge effects, may also explain the nonsignificant findings.

Regarding the retention of declarative memory content, the differences between the suppression conditions were negligible. Previous studies provide solid evidence that SWS, sleep spindles, and their synchronized activity have significance in declarative memory consolidation (Plihal and Born, 1997, 1999; Clemens et al., 2005; Helfrich et al., 2018; Léger et al., 2018; Klinzing et al., 2019; Mikutta et al., 2019; Muehlroth et al., 2019; Halonen, Kuula, Antila, et al., 2021; Halonen et al., 2022). In the current study, while SO–spindle coupling events were markedly reduced via SWS suppression, this did not result in statistically significant retention differences relative to intact SWS (suppressed REMS). This suggests that the impact of (SO-coupled) sleep spindles during SWS in memory consolidation is not dose-dependent. In line with this, another SWS/REMS suppression study reported no declarative memory difference between the conditions (Wiesner et al., 2015). The authors proposed that the residual sleep was sufficient to consolidate the memorized items. Indeed, the amount of SWS may not be consequential for memory retention, as a review concludes (Cordi and Rasch, 2021).

A previous study with SWS/REMS deprivation found that N2 spindles predicted verbal learning (Genzel et al., 2009). Thus, we expected that N2 spindle/SO–spindle activity would “compensate” for the suppressed SWS events, but no evidence on this was observed. A possible explanation for the lack of associations and intercondition differences may be that memory consolidation is not dependent on a single sleep stage. According to the sequential hypothesis (Giuditta, 2014), novel memories are processed and consolidated across successively occurring SWS and REMS episodes. Such intact duets were absent in our study. Experimental evidence suggests that establishing new semantic schemas is dependent on REMS that follows SWS (Batterink et al., 2017). Thus, in our study, consolidating novel semantic metaphors may have been disrupted by suppressing either SWS or REMS, not only SWS.

We found that NREMS oscillations predicted overall (including presleep) recall performance for declarative memory content. Specifically, fast spindle density during N2 associated with better memory outcome across the whole sample. Sleep spindle activity possibly reflects learning ability beyond memory consolidation, as evidenced in earlier studies (Gais et al., 2002; Berner et al., 2006). Both encoding and retrieval require coordinated activity of the thalamus and neocortex (Staudigl et al., 2012; Wagner et al., 2019), and the properties of these structures and their white matter connectivity impact the generation and propagation of spindles (Piantoni et al., 2013; Fernandez and Lüthi, 2020). NREMS oscillations may thus mirror the capacity for initial learning and successful memory retrieval (Staudigl et al., 2012; Wagner et al., 2019).

Strengths of our study include a comparative paradigm with suppressing both REMS and SWS, enabling the examination of both the affective and mnemonic impact of disrupted sleep. Additionally, measuring oscillatory activity enabled investigating the (dose-dependent) relevance of the modulated activity on off-line processing.

Some limitations must also be addressed. First, not having a control condition with undisturbed sleep prevented us from observing nonspecific impacts of disrupted sleep on stress reactivity, and thus, generalizing the results to undisturbed sleep should be done with caution. However, the results converge with a previous study, where the amount of undisrupted REMS predicted higher postsleep SCR toward a similar stressor (Halonen, Kuula, Makkonen, et al., 2021). Additionally, none of the participants had intact sleep cycles. Suppressing either SWS or REMS disrupts sequential off-line processing (Giuditta, 2014), preventing the complementary influence of these sleep stages on the establishment of new memory schemas (Batterink et al., 2017). Second, the sample size was small, limiting statistical power and increasing the possibility of false findings (Button et al., 2013). Especially between-condition comparisons were low-powered, which may have led to an inadequate account of the suppression effects. Third, we did not have a control stimulus for affective response. It remains unclear whether the observed response was specific to own-singing playback, i.e., self-conscious affect. However, in a previous study (Wassing et al., 2019), REMS-driven affective modulation was specific to participants’ own singing, and not to a professional singer's performance. Finally, the suppression conditions differed in singing experience and number of awakenings. While optimally such parameters should be matched between conditions, statistically controlling for their effects on SCR did not change the outcome.

## Conclusions

This study contributed to understanding of emotional and declarative off-line processing in several ways. Selective suppression of REMS or SWS suggests that the physiological response toward self-conscious emotional stressor is preserved when REMS remains intact and SWS is disrupted and that REMS theta activity likely facilitates the preservation of the affective charge of the memory. The retention of declarative memories was not impaired by suppressed SWS to any greater extent than by suppressed REMS, suggesting that off-line memory consolidation is not dose-dependently related to NREMS oscillations only.

## Data Accessibility

Research materials are available from the corresponding author on reasonable request. The scrips used to create study variables were implemented and executed with Python 3.9 or MATLAB R2022b. The scripts are found as Extended Data Figure 1-1 and are available on https://version.helsinki.fi/sleep-mind/karaoke-2024.

10.1523/ENEURO.0453-23.2024.d1 Data 1 Download Data 1, ZIP file.

## Synthesis

Reviewing Editor: Frederike Beyer, Queen Mary University of London Decisions are customarily a result of the Reviewing Editor and the peer reviewers coming together and discussing their recommendations until a consensus is reached. When revisions are invited, a fact-based synthesis statement explaining their decision and outlining what is needed to prepare a revision will be listed below. The following reviewer(s) agreed to reveal their identity: Hanna Isotalus.

As shown in the comments below, both reviewers judge the work presented here as of interest to the field and generally high qualtiy, but make important suggestions regarding the presentation and discussion of results, as well as some additional analyses to include. Both reviewers have provided very detailed feedback, which should be addressed in the revision of this manuscript.

Best wishes,

Frederike

Reviewer 1 comments:

This paper examined the role of SWS and REM in affective retention and memory using a sleep stage suppression study design in humans. The authors found that intact REM (SWS suppression) resulted in an increased affective response the next day. They further reported that REM theta activity was positively correlated with changes in skin conductance response. This paper addresses a critical gap in the literature; however, it could benefit significantly from clarification in the writing, additional spindle analyses, and most importantly, a thorough discussion on the difference between subjective v objective embarrassment across the night and its relation to REM. We believe the authors should be given the opportunity to respond to these comments and edit the manuscript for future review. Our comments per section are below:

Introduction

1. This is not a sleep specific journal, readers would benefit from additional background on NREM/REM, linking SWS to NREM right away, SO-spindle coupling, etc.

2. It would benefit the reader to have greater detail on the theory tested (Walker Sleep to Remember/Forget) in the introduction. It is briefly mentioned, without additional details, but a clear critical aspect of the paper. Forgetting is a key part in the memory process as well and should also be better explained in the introduction.

3. A mechanism not discussed is tagging emotional information during encoding for preferential memory processing (i.e. Denis et al., 2022; Payne &Kensinger, 2018). This seems relevant to the current paper and should be discussed.

4. Generally, few details on the directionality of relationships are discussed throughout the paper, making it at times unclear what is being stated. For example, in the paragraph lines 74-84, it is unclear what the relationships are between REMS and theta activity with freezing behavior and affective regulation. Given the last line of the paragraph, it also appears as if the results are contradictory. This is one example, but throughout the manuscript, more details of studies and directionality of results are needed.

5. There are a number of SO-spindle coupling papers published on sleep-dependent memory consolidation, however, the text implies otherwise. These should be cited and this should be corrected.

6. Can the authors include reminders throughout the writing that SWS suppression is intact REM and vice versa. This will help the reader while reading through the papers

Methods:

1. Minor Comments:

o Age breakdown is not included in methods section

o Timing in methods paper flips between military time and standard

o N4 is not used in AASM, it's unclear why this is referenced on line 134.

o Arousal measurements (types and lengths of time it took to arouse individuals) should be added per condition

o Were subjective embarrassment ratings or SCR measured pre/post karaoke or during? Additionally, the timing of these measurements could be added to Figure 1 for clarity.

2. Was memory retrieval and karaoke playback order counterbalanced across days? Order is unclear (across subjects/days). It seems as though, during day 1, playback is presented before memory retrieval 1, yet day 2 memory retrieval occurs before the second playback. This order may influence memory retrieval as previous work has found that stress can inhibit episodic memory retrieval (see Shields et al., 2017; Schwabe et al., 2012).

3. What was done with EEG bursts referenced in line 191/192. Were they excluded from the data?

4. The calculation for REM suppression is slightly confusing, did you not include SWS?

5. What of the night before sleep? No results in the zmax were discussed except for demographic table? Did this influence baseline results.

Results:

1. As mentioned before, overall, the results need to include the direction of the effects and more details explaining the meaning of the finding.

2. Table 1 is unclear, although it matches the figure, it would benefit the readers to call night 1 - baseline and night 2 experimental, or some such thing. As is, it becomes confusing if night 1 is actually referenced to the suppression night (occurring the night of day 1).

3. The subjective embarrassment result should be included in the main manuscript, rather than the supplemental. Subjective embarrassment should be considered overnight subjective affective. In this case, subjective affect decreased overnight. SCR may objectively show increased affect, yet in this case subjective and objective affect vary in direction. This should be considered and discussed in the manuscript. Studies in line with the SFSR hypothesis typically study affective response through subjective reports of emotions or fMRI reactivation of the amygdala. In this case, your subjective results follow this hypothesis and past literature.

4. In line with point 3 above, was there any memory aspect of the karaoke experience? If not, the SFSR hypothesis is specifically about emotional memories and the separation between the emotional reactivity and the memory of the event. If memory was not tested, this should be clarified in the manuscript text.

5. It would be beneficial to add more details on overnight memory change for metaphor recall.

6. I disagree with the analysis of only a 'fast' spindle frequency. The current analyses are done under the assumption that there will be two peak modalities, a slower and faster range, within the sigma frequency. When computing analyses in this manner, the data is biased to fit these two modalities. The spindle data should to be reanalyzed using the broad band of the sigma frequency. If two peaks are shown to exist, without apriori separation, this should be reported and then further analyses should be conducted on both fast and slow spindles. However, if a bimodal peak is not found and as the literature does not always differentiate between slow and fast spindles when investigating sleep-dependent memory consolidation, analyses should be re-run with the entire spindle range (see Klinzing et al., 2019)

Discussion:

1. Please comment on subjective embarrassment rating results and how those results fit into the context of prior literature (see comments above).

2. Greater details on the results of the Spoormaker et al., 2012 and Halonen et al., 2021 studies are needed. Where does this result fit into the context of those two previous studies. Please elaborate on this point.

3. Recent work suggests greater improvements on NREM-dependent tasks when followed by REM sleep, compared to sleep without REM (Batterink et al., 2017; Singh et al., 2022). It may be possible that declarative memory consolidation requires a full sleep cycle, yet these findings are not mentioned in the discussion. This manuscript may benefit from discussing this.

Print Report

Reviewer 2 comments:

The authors have submitted an interesting manuscript, looking into the effects of selective sleep stage deprivation on emotional processing and declarative memory. The protocol of the study is well designed with appropriate measures being used, with some limitations on the chosen analysis methods. The main finding of the study is that there is no overall difference in emotional processing (as measured by skin conductance) or declarative memory following either sleep stage suppression, but the paired difference from before-to-after-sleep emotional processing is increased following slow wave, but not REM suppression. This increase in SCR was correlated with REM theta and SWS alpha power. This finding is interesting.

The manuscript could be improved by a stronger and more clearly written result section. There are some omissions in the result section. E.g. results and details of power calculation, details of how many tests were done and what the main findings were, specific test results such as test statistics, degrees of freedom, sample sizes for each test, specific p-values, and details of which results are corrected for multiplicity and which not, are missing. Many of the results do not mention directionality (e.g. lines 341 &369). These details should be included in the core text of the manuscript. This section could overall do with some clarifications as well. For example, sentences summarizing main results of each section at the beginning would help with readability. It would also benefit from clarifying which analyses were planned and which were exploratory.

Extended data table 1-1 should be included in the core text. It should also include multiplicity correction.

Below are comments section-by-section. Please note that while there are many comments these are mostly minor things that the authors can clarify without needing to do further analyses, or clarifications on the points made above.

Abstract:

- include participant age, clarify n for each experiment

- include effect size and some information on type of tests carried out.

- Include main finding that there was no overall difference in SCR between REM and SWS suppression.

- consider use of the word 'significant', especially in absence of explaining what this means (i.e. providing stats). For a more thorough discussion on why this language isn't encouraged, see e.g. the 2019 comment in Nature by Armhein et al ("Scientists rise up against statistical significance"). Related to this point, on line 31-32 the abstract sentence beginning "Intact SWS...." makes a claim not supported by the proof in this manuscript. Absence of evidence is not evidence of absence. Please consider this comment when editing the results section.

- Line 30: Is "especially" the right word here?

- This abstract could do with a stronger conclusion about what the results mean. The loose link to mental health should either be omitted or justified by linking these two more strongly in the discussion

Significance statement:

- Given REM suppression did not impact on SCR, the statement that "We show that rapid eye movement sleep [is] essential for preserving the affective response overnight" did not align with my interpretation of the result. Better to focus on the REM theta and impact of SWS suppression. This finding is novel and interesting.

Introduction

- lines 55-56 : many of the aforementioned /referenced studies are also experimental, and therefore experimental studies have been performed.

- Line 71-73: Could you re-word this sentence as it IMPLIES that the next paragraph will explain sleep suppression studies in rodents, even though (at least some of) those studies are not sleep suppression protocols. Alternatively clarify in the next paragraph, that these are not sleep suppression studies.

- Paragraph starting line 74: specify what is meant by "rodents"

- Line 93-94: Helfrich et al, 2018 link spindle-SO coupling with memory during stage 3, similarly Weiner et al 2022.

- Final paragraph: State hypotheses clearly to clarify which analyses were planned and which were exploratory.

Materials and Methods

- State participant age, central tendency and spread in text

- Line 177-178: Include short sentence explaining rationale of using SCL or SCR (i.e. what different information do they convey, how should they be interpreted differently by the reader) either here or elsewhere in the manuscript.

- Line 181: mean of what? Is this the mean rating of the two questions or of all trials?

- Line 134 says SWS was interrupted targeting N4 stage specifically, but then on line 190 the authors report using AASM for scoring stages N1, N2, N3 and REM, and refer to N3 as SWS. Please clarify why N4 was used in experimental protocol but was not used in scoring. Please report which version of AASM was used to score as it is not clear if it is a version that includes N4 or not. Which protocol was used for identifying stage 4? Somewhere in manuscript or supplementary, include a clear outline of which analyses relate to N2, N3 and N4.

- Line 300: Explain definition of "trend level". This appears to be defined by a p-value that doesn't surpass the set alpha threshold. Give clear rationale of why significance is defined as alpha 0.05 and how that relates to "trend-level". When discussing trend-level, include details of effect size and ideally plots (even if in SM)

- Line 301: have authors considered or tried using another, less conservative, correction method for multiple testing? There are many tests and something like the Benjamini-Hochberg protocol may be more appropriate for family-wise corrections

- Line 304: provide details of the power analysis, including how it was conducted, what the suggested alpha and beta were and which literature guided it. Including whether the studies referred to were between or within subjects designs. Include a short rationale. This study appears under powered for this number of analyses.

Results

- Consider whether using the word 'significant' so heavily when the data sample is so small is a reasonable approach. This section would be stronger with a heavier reliance on the effect sizes and description of the data.

- It appears that the most important result discussed here is that the difference in SCR when subtracting Day 1 and Day 2 scores differed between SWS and REM, but no such difference is seen in the difference between Days 2 and 5. Was this a planned analysis? From Fig 3b, it appears that this difference is driven by Day 2 SCR, but the plot does not show the paired nature of the time-data, so it is difficult to assess this. The paper should have a paired difference plot to compliment this important analysis. Further, with small sample size, outliers and data that appears non-parametric, the use of parametric tests should be justified.

- Throughout, please provide details of statistical tests including test statistics, not just p-values. Include degrees of freedom and central tendencies (with spread). Give details of the type of statistical test conducted. Provide details of the type of multiplicity correction used and report corrected p-values for each test.

- The manuscript should include a time-by-time comparison of SCR (i.e. same as extended dataplot 3-2 but for SCR as opposed to subjective measures).

- Provide details of all the tests that were carried out regardless of whether they were significant or not.

- Line 332: Clarify here that "condition" means no difference between SWS and REM suppression. This would aid readability of the entire results section, e.g. also line 342.

- Line 347: Clarify this sentence, explain the interaction. Also line 351.

- Line 351: Give more detail about the trend (e.g. a plot) - what makes this trend-level?

- There appears to be a lack of consistency on the reporting in the figures, where in some * refers to a result having been Bonferroni-corrected, but in other results figures it refers to specific p-values. Please clarify the figures and make them consistent. Clarify in figure 3 what the * means - are these all Bonferroni-corrected?

- Plotted data does not appear to be normally distributed, yet all statistical tests used assume normal distribution. Q-Q plots, assuming they're plotted against normal distribution, are clearly not showing a good fit. How was normality of data assessed? Consider non-parametric tests, especially given small sample sizes. This would also allow for not excluding the outliers, unless this is due to measurement error. Figure 3c: Please provide a clearer figure legend to explain this figure, how do the many stars relate to the p-value and Bonferroni correction?

- Figure 3b: I do not think a barplot is the best plot to represent this data, as it gives an impression that the height is of importance, but in these data the central tendency is more important . The data would be easier understood with a more appropriate plot, e.g. a boxplot or a violin plot. Consider also plotting the individual data points so they are more clearly visible.

Discussion:

- Line 399: Does this refer to the result reported on line 336? It is not clear how and what the analysis comparing SCR between SWS and REM suppression was carried out. It is stated on line 332 that there is no main effect. Could the difference here, as one of the reported main results of the paper, be plotted as a figure

- Line 406: does this refer to the Day 1 minus Day 2 difference? Is there a difference on Day 2 SCR between SWS and REM suppression? I cannot find this in the results section and it is not clearly laid out. Please show the day-by-day analyses comparing SCR (and not just difference) clearly after the paragraph starting on line 322 of results. These paired analyses should be reported and plotted.

- Line 460: "this did not affect the memory retention result overnight" - absence of evidence is not evidence of absence. Lack of a significant effect doesn't mean there was no effect. Either re-word to keep interpretation to the analyses provided, or provide analyses to quantify the certainty of no effect (e.g. Bayesian approaches can quantify the likelihood of no effect, but parametric tests shown here do not).

- Line 491: I agree that statistical power of this study is limited and I think this should be highlighted earlier in the discussion. This section should also elaborate on the between-subjects design and re-instate what the sample size is. There was not sufficient detail given on the power calculation in this manuscript, but how does the sample size contrast to the power calculation and samples in similar studies? E.g. most other studies use a within-subjects design which should also be discussed - having 30 participants in a within subjects design is more powerful than 30 participants in a between subjects design, and without detail of the power calculation, it is unclear if this was considered when deciding on sample size. This section should also discuss why many of the tests were not corrected for multiple comparisons, and whether the main finding was an exploratory or planned analysis.

- Line 505: I agree with the conclusion that this has tangential relevance to mental health. However, this point should also be elaborated on earlier in the discussion.

Minor / typographical and grammatical observations

- Line 58: typo: "In humans" or "in human studies" but not "in humans studies"

- Line 60: typo: "habituation" not "the habituation"

- Line 129: great choice of song

- Line 131: at ~21 what? Hours?

- Line 131: typo overwent to underwent

- Line 132: setup rather than installed

- Line 188: typo: "on" rather than "in" the forehead

- Line 198: Please use consistent nomenclature - i.e. use REM or REMS but not both in one manuscript

- Line 300: Typo: alpha = 0.05 as opposed to p<.05

- Line 304: Typo: a priori - as opposed to apriori

- Line 328: Sentence starting on this line is grammatically unclear

- Line 362-64: Clarify this sentence.

- Line 393-95: Clarify this sentence.

- Line 463: "in line"?

- Line 504: Clarify this sentence.
