# Supplementary material for: REM Sleep Preserves Affective Response to Social Stress—Experimental Study
Source: eNeuro. 2024 Jun 3;11(6):ENEURO.0453-23.2024. doi: 10.1523/ENEURO.0453-23.2024 (PMC11151192; doi:10.1523/ENEURO.0453-23.2024)
Supplement: Table 2-1 — The effects of suppression condition and power spectral density values on emotional response values/change in non-parametric statistics. Download Table 2-1, DOC file. [file eneuro-11-ENEURO.0453-23.2024-s003.doc]

| Extended Data Table 2-1. The effects of suppression condition and power spectral density values on emotional response values/change in non-parametric statistics. | | | | | | | | | |
| --- | --- | --- | --- | --- | --- | --- | --- | --- | --- |
|  |  | Skin conductance response | | Subjective embarrassment | |  |  |  |  |
| Friedmann's test | | χ2 | p | χ2 | p |  |  |  |  |
|  | All | 26.690 | <0.001*** | 30.897 | <0.001*** |  |  |  |  |
|  | REMS_SUPPR_ | 9.733 | 0.046* | 25.200 | <0.001*** |  |  |  |  |
|  | SWS_SUPPR_ | 19.000 | <0.001*** | 8.714 | 0.077 |  |  |  |  |
|  |  |  |  |  |  |  |  |  |  |
| Mann-Whitney U | | Z | p | Z | p |  |  |  |  |
|  | Day1 | -0.567 | 1.000 | -0.745 | 1.000 |  |  |  |  |
|  | Day2 | -0.567 | 1.000 | -1.011 | 1.000 |  |  |  |  |
|  | Day3 | -0.262 | 1.000 | -0.332 | 1.000 |  |  |  |  |
|  | Day1-to-Day2 | -2.575 | 0.016* | -0.044 | 1.000 |  |  |  |  |
|  | Day2-to-Day5 | -0.611 | 1.000 | -2.078 | 0.078 |  |  |  |  |
|  |  |  |  |  |  |  |  |  |  |
|  |  | Day1-to-Day2 SCR | | Day2-to-Day5 SCR | | Day1-to-Day2 embarrassment | | Day2-to-Day5 embarrassment | |
| Spearman's correlation | | r | p | r | p | r | p | r | p |
| REMS | |  |  |  |  |  |  |  |  |
|  | Delta | 0.550 | 0.012* | -0.232 | 1.000 | -0.192 | 1.000 | 0.299 | 0.690 |
|  | Theta | 0.525 | 0.018* | -0.118 | 1.000 | -0.305 | 0.648 | 0.328 | 0.498 |
|  | Alpha | 0.460 | 0.072 | -0.077 | 1.000 | -0.171 | 1.000 | 0.308 | 0.630 |
|  | Sigma | 0.368 | 0.300 | -0.068 | 1.000 | -0.104 | 1.000 | 0.264 | 0.996 |
|  | Beta1 | 0.319 | 0.552 | -0.053 | 1.000 | -0.057 | 1.000 | 0.401 | 0.186 |
|  | Beta2 | 0.235 | 1.000 | -0.035 | 1.000 | 0.106 | 1.000 | 0.335 | 0.456 |
| SWS | |  |  |  |  |  |  |  |  |
|  | Delta | 0.006 | 1.000 | -0.122 | 1.000 | -0.087 | 1.000 | -0.249 | 1.000 |
|  | Theta | 0.066 | 1.000 | -0.126 | 1.000 | -0.135 | 1.000 | -0.267 | 0.966 |
|  | Alpha | 0.063 | 1.000 | -0.106 | 1.000 | -0.073 | 1.000 | -0.260 | 1.000 |
|  | Sigma | 0.067 | 1.000 | -0.176 | 1.000 | -0.116 | 1.000 | -0.322 | 0.528 |
|  | Beta1 | 0.056 | 1.000 | -0.190 | 1.000 | -0.173 | 1.000 | -0.429 | 0.120 |
|  | Beta2 | -0.007 | 1.000 | -0.210 | 1.000 | -0.183 | 1.000 | -0.469 | 0.060 |
| Friedman’s test: repeated measures test across Day1, Day2 and Day5. Mann-Whitney U: comparison of the suppression conditions. SCR: skin conductance response. REMS: rapid eye movement sleep. SWS: slow wave sleep. *** p<.001, * p<.05, Bonferroni-corrected. | | | | | | | | | |
